# Dietary Flaxseed Oil Protects against Bleomycin-Induced Pulmonary Fibrosis in Rats

**DOI:** 10.1155/2012/457031

**Published:** 2012-08-08

**Authors:** Joshua Lawrenz, Betty Herndon, Afrin Kamal, Aaron Mehrer, Daniel C. Dim, Cletus Baidoo, David Gasper, Jonathan Nitz, Agostino Molteni, Richard C. Baybutt

**Affiliations:** ^1^Department of Applied Health Science, Wheaton College, 501 College Avenue, Wheaton, IL 60187, USA; ^2^Department of Basic Medical Sciences, School of Medicine, University of Missouri-Kansas City (UMKC), USA; ^3^Department of Pathology and Pharmacology, School of Medicine, University of Missouri-Kansas City (UMKC), 2411 Holmes Street, Kansas City, MO 64108, USA; ^4^Department of Anesthesiology, School of Medicine, University of Missouri-Kansas City (UMKC), Kansas City, MO 64108, USA

## Abstract

Bleomycin, a widely used antineoplastic agent, has been associated with severe pulmonary toxicity, primarily fibrosis. Previous work has shown a reduction in bleomycin-induced lung pathology by long-chain omega-3 fatty acids. Treatment by short-chain omega-3 fatty acids, **α**-linolenic acid, found in dietary flaxseed oil may also reduce lung fibrosis, as previously evidenced in the kidney. To test this hypothesis, 72 rats were divided between diets receiving either 15% (w/w) flaxseed oil or 15% (w/w) corn oil (control). These groups were further divided to receive either bleomycin or vehicle (saline) via an oropharyngeal delivery, rather than the traditional intratracheal instillation. Lungs were harvested at 2, 7, and 21 days after bleomycin or saline treatment. Animals receiving flaxseed oil showed a delay in edema formation (*P* = 0.025) and a decrease in inflammatory cell infiltrate and vasculitis (*P* = 0.04 and 0.007, resp.). At days 7 and 21, bleomycin produced a reduction in pulmonary arterial lumen patency (*P* = 0.01), but not in rats that were treated with flaxseed oil. Bleomycin-treated rats receiving flaxseed oil had reduced pulmonary septal thickness (*P* = 0.01), signifying decreased fibrosis. Dietary flaxseed oil may prove beneficial against the side effects of this highly effective chemotherapeutic agent and its known toxic effects on the lung.

## 1. Introduction

Bleomycin is a group of glycopeptides that binds iron and oxygen in vivo to produce an active drug, effective in cancer treatment. In the last few decades, many Americans were diagnosed with Hodgkin's lymphoma and testicular cancer, and a majority received bleomycin as part of their chemotherapeutic regimen. A large study from 1986 to 2003 found that out of 141 Hodgkin's lymphoma patients treated with bleomycin, 18% developed pulmonary toxicity, and of those patients 24% died [1]. Currently, no known treatments exist to prevent pulmonary toxicity in these patients. In short, bleomycin's fibrotic side effects are so common that it is widely used to create animal models of pulmonary fibrosis.

Bleomycin's active intermediate is believed to induce both single-and double-strand DNA cleavage in neoplastic cells [2]. The chemotherapeutic mechanism results from the chelation of iron ions with oxygen, which leads to production of DNA-cleaving superoxide, and also hydroxide free radicals [3–5]. It is the increased production of reactive oxygen species (ROS) that may be critical in producing proinflammatory eicosanoids that lead to bleomycin's pulmonary toxicity, and may eventually lead to lung fibrosis [6–9]. In recent literature, the presence of several ROS has been found in clinical cases of idiopathic pulmonary fibrosis [10, 11], and decreased production of ROS has been shown to protect mice against bleomycin-induced pulmonary fibrosis [12]. In addition, a reduction in antioxidants has been reported in IPF lungs, and the resulting oxidant-antioxidant imbalance has been suggested in the progression of IPF [13, 14]. 

Though it still remains unclear the role which oxidation plays in the inflammatory and profibrotic response found in pulmonary fibrosis, oxidative stress seems to be associated with the disease as previously described. As a means of attenuating oxidative damage, long chain omega-3 fatty acids, eicosapentaenoic acid (EPA), and docosahexaenoic acid (DHA) have been found effective due to their protective antioxidant properties [15, 16]. In addition, we have previously shown that fish oil containing EPA and DHA protects against lung inflammation and pulmonary fibrosis in a monocrotaline-induced lung fibrosis model [17]. Another research group has shown that fish oil prevents bleomycin-induced lung inflammation and pulmonary fibrosis [18]. Thus, the long chain omega-3 fatty acids, eicosapentaenoic acid (EPA) and docosahexaenoic acid (DHA), are thought to be responsible for these protective effects. In addition, the essential omega-6 fatty acid, *γ*-linolenic acid (GLA) has also been shown to be a potent antioxidant [19], and has been found to attenuate bleomycin-induced lung fibrosis in hamsters [20]. Whether short chain omega-3 fatty acids have a similar protective effect is not known. 

Shorter-chain omega-3 fatty acids such as *α*-linolenic acid (ALA) in flaxseed oil do not necessarily have similar biological effects as the longer chain omega-3 fatty acids found in fish oil. For example, long chain fatty acids EPA and DHA have been shown to have cardioprotective effects, while the short chain fatty acid ALA did not reduce or benefit cardiovascular disease outcomes in a recent review [21]. Recent studies also found that low doses of fish oil (0.7–7% energy), not flaxseed oil, suppressed inflammation, as evidenced by decreased thromboxane B_2_ and serum TNF-*α* levels, markers for inflammation [22]. On the other hand, a recent study indicates that dietary ALA in rapeseed oil has antioxidant properties, as it inhibits lipid peroxidation in animals after acute brain ischemia [23]. Also, dietary ALA has been shown to elicit an anti-inflammatory effect in cultured peripheral blood mononuclear cells by inhibiting the proinflammatory cytokine production of IL-6 and TNF-*α* [24]. Furthermore, a research group at the University of Manitoba has found substantial evidence that dietary flaxseed oil protects against fibrosis in the kidney [25–29]. 

Therefore, there seem to be similarities and differences in the biological function of the shorter chain omega-3 fatty acid, ALA found in flaxseed oil, and its close relatives GLA, EPA, and DHA. All have been shown to have antioxidant properties, and both anti-inflammatory and antifibrotic effects under certain conditions. To our knowledge, no one has investigated whether ALA found in dietary flaxseed oil protects against bleomycin-induced lung fibrosis. This is the purpose of the present study. 

## 2. Materials and Methods

### 2.1. Animals and Treatment

Weanling male Harlan Sprague-Dawley rats (Indianapolis, IN, USA) were housed in stainless steel cages at approximately 24°C with a 12-hour light-dark cycle. Animal care and use were approved by the Institutional Animal Care and Use Committee (IACUC) of Wheaton College. Animals were housed in an animal facility approved by the American Association for the Advancement of Laboratory Care (AALAC). The rats had ad libitum access to food and water.

A total of 72 rats weighing 40–60 g each were randomly assigned to one of four groups (18 rats per group). All rats were fed a standard AIN-93G diet [30] containing either corn oil (15% w/w) or flaxseed oil (15% w/w). The antioxidant activity of tert-Butylhydroquinone (TBHQ), an effective preservative for unsaturated oils, was not significantly different than that of the small amounts of natural tocopherols found in the corn oil and was substantially less than the amount used in studies that evaluated the antioxidant role of TBHQ [31]. To minimize fat oxidation, the powdered diets were stored in Ziploc freezer bags, and stored at −20°C. The diets were purchased from Dyets Inc., (Bethlehem, PA,). Food consumption was measured by calculating the difference between the preweighed and unconsumed diet. Food was provided daily and the leftovers discarded. Body weights were recorded every week.

After four weeks of dietary treatment of the respective diets, bleomycin was administered. About 5 mL of Trifluralin anesthesia was used to saturate a piece gauze placed in a bell jar. Rats were placed in the covered jar became anesthetized after five seconds of gaseous exposure to Trifluralin, and remained anesthetized for about 30 seconds. During this time, bleomycin or its vehicle (saline) was administered oropharyngeally in a 400 *μ*L solution (8 U/kg body weight) to half of the rats in each respective group, according to a previously published method [32]. The tongue was secured by tissue forceps in such a way as to prevent swallowing, and the rats aspirated the bleomycin solution that was administered via pipette into the oral cavity. The four treatment groups consisted of rats were instilled with the vehicle and fed a corn oil diet (VC), the vehicle and fed a flaxseed oil diet (VF), bleomycin and fed a corn oil diet (BC), and bleomycin and fed a flaxseed oil diet (BF). At the termination of the experiment, blood was collected from the rats, followed by organ removal of the lungs, liver, kidneys, and heart at 2, 7, or 21 days after bleomycin administration.

### 2.2. Assessment of the Lung Histological Damage

Histological evaluation of lung tissue was performed in a semiquantitative manner as previously described [33–36]. Briefly, the left lung was removed and then prepared with 10% buffered formalin, and fixed for 1 week. The right lung was immediately frozen in liquid nitrogen and stored at −80°C for analysis. Formalin-fixed lungs were then embedded in paraffin blocks and sections were prepared for hematoxylin eosin staining and Masson-Trichrome collagen staining. For evaluation of pulmonary damage, slides were scored by two pathologists who were unaware of the experimental protocol and their scores were averaged to obtain a single score. Observed changes were evaluated in the thickening of the alveolar septa, and interseptal, intra-alveolar, and vascular areas were examined for the presence of hemorrhaging, inflammatory cells, or collagen deposition (fibrosis). 

Particular evidence was given to the fibrosis (collagen deposition) expressed in the vasculature, the septa, and the peribronchial musculature. Subjective scoring ranging from 5 (presence of borderline damage) to 40 (very severe and extensive damage with destruction of a large portion of the parenchyma) was assigned to the components of the lung previously stated. The total score reported was the mean ± standard error of the mean of the individual scores for each category. Values for each group of treated animals were averaged and reported separately.

The method for determining lumen patency and media/adventitia ratio has been previously published in different models of lung injury including exposure to radiation, development of pulmonary hypertension and fibrosis, and damage induced by vitamin A deficiency [33–38]. Briefly, the luminal diameter of vessels divided by their external or vascular diameter was the measurement associated with lumen patency. The outer diameter of the media divided by the outer diameter of the adventitia provided the media/adventitia ratio, which was used as a measure of adventitial edema. Both ratios were evaluated in five small caliber pulmonary arteries for each rat in each group. The mean value of the five vessel measurements for each rat was reported for statistical analysis. The thickness of the arteriolar wall and the percentage of small artery lumen occlusion (diameter ranges from 20 to 100 *μ*m) were measured in photographs at 100X and 400X magnifications. 

### 2.3. Statistical Analysis

Data were expressed as means ± SEM. The experiment was analyzed as a 2 × 2 factorial design. Differences among groups for inflammatory cell infiltrate, septal thickness, emphysema, medial vasculitis, and edema were determined using a two-way analysis of variance (ANOVA) followed by least significant difference (LSD) multiple comparison tests (SAS Institute Inc., Cary, NC, USA). For the semiquantitative analysis, group data were compared by a one-way analysis of variance with Tukey posttest. These were calculated using StatView software. The level of significance was *P* < 0.05 for all comparisons.

## 3. Results

### 3.1. Morbidity and Mortality

No rats died during the four-week dietary treatment prior to bleomycin treatment. Four rats died during the administration of bleomycin or its vehicle due to too much exposure to anesthesia. These four rats were excluded from statistical analyses. Six rats died after bleomycin treatment and before their sacrifice date. Two BC rats died on day 3 (after bleomycin treatment) and one BC rat died on day 8. Two BF rats died on day 2 and one BF rat died on day 8. Localized darkening areas (hemorrhaging) were observed on the lung surfaces in rats that died on day 8. Otherwise, all rats survived and were sacrificed at 2, 7, or 21 days after bleomycin treatment. 

### 3.2. Body Weight Gain and Food Intake after Bleomycin Treatment

**Figure 1:**
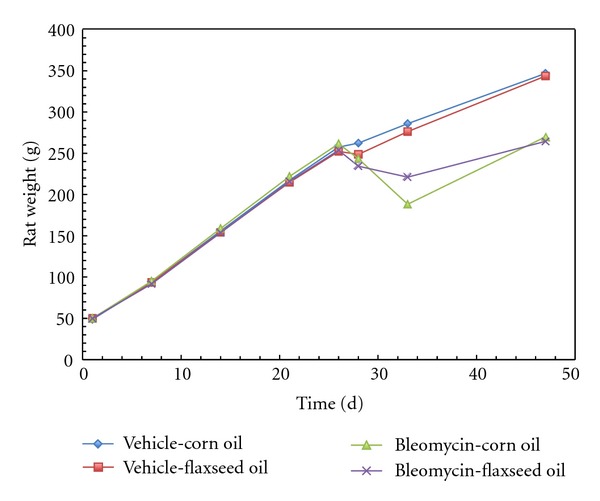
Weight gain in rats fed 15% (w/w) corn oil or 15% (w/w) flaxseed oil diet. Bleomycin and vehicle administration occurred at day 26. Data are expressed as means ± SEM, *n* = 13–15. At day 33 (or 7 days after bleomycin administration), there was significantly less weight loss in the BF treated rats than the BC treated rats (*P* < 0.03).

The average daily food intake for the bleomycin-treated rats was significantly less than the vehicle group (*P* < 0.03). The food intake of the BF group was significantly more than the BC group (*P* < 0.001). There was also significantly less weight loss in the BF-treated rats than the BC-treated rats at 7 days (*P* < 0.03). The findings are summarized in Table 1 and Figure 1.

### 3.3. Gross Organ Evaluation

At gross evaluation, the vehicle-treated rat lungs (VC and VF) at 2 days, 7 days and 21 days appeared to be normal. Discoloration, or localized darkening areas (hemorrhaging) and a cobblestone appearance were observed in the lungs of BC rats at 7 days, and to a more severe extent at 21 days. There were no abnormal damages on the rat lung surfaces at 2 days. In contrast, bleomycin-treated rats fed a flaxseed oil diet were seen to have less organ discoloration and surface hemorrhaging. Other organs appeared similar to the controls. 

### 3.4. Organ Tissue Weights

The relative weights of the respective organs of bleomycin-treated rats were all significantly higher when compared to the organs of the vehicle controls (*P* < 0.001 for all but liver where *P* < 0.002). The average relative weights of all five of the organs (lung, liver, heart, left kidney, and right kidney) in the BF treated rats were significantly less than that of the BC-treated rats at 7 days (*P* < 0.05). The findings are summarized in Table 2.

### 3.5. Overview

**Figure 2:**
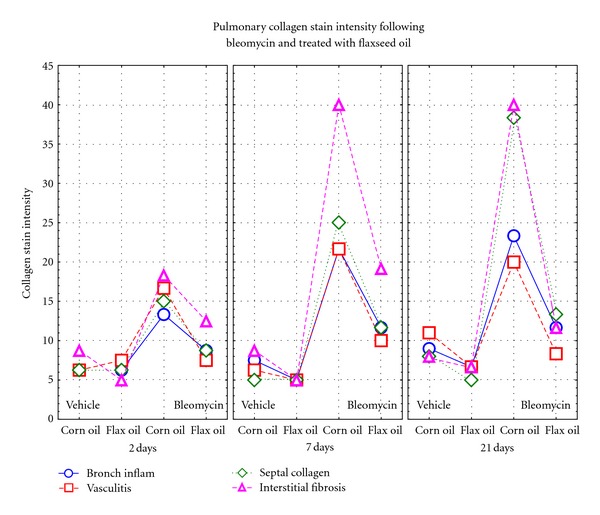
Qualitative evaluation of the pathological scores of the various treatment groups at each time interval point. (Staining: Masson's trichrome). The symbols represent the mean of all the rats in each group. Scoring of pathology: Ranges from 5: borderline damage, progressing to a high of 40: very severe and extensive damage. Scoring of stain intensity: estimated area of blue stain on a series of high-power fields with trichrome staining. Abbreviations: Bronch. Inflam.: Bronchial Inflammation with loss of epithelium, loss of cilia, and increased number of inflammatory cells. Vasculitis: Thickening  of the media of vessels and reduction of lumen patency. Septal collagen: Presence of blue stained fibers on trichrome stain. Interstitial fibrosis: Presence of fibers in the septa stained by trichrome.

**Figure 3:**
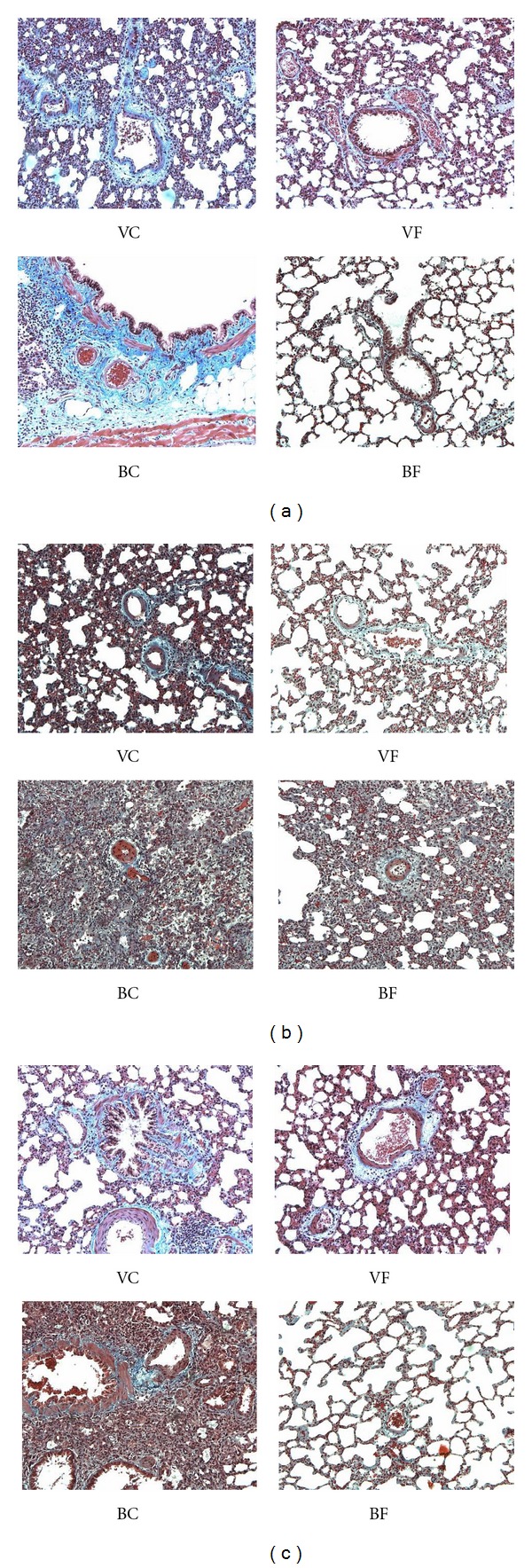
(a) Histological sections of rat lungs of the four groups sacrificed at day 2. Mild septal congestion and bronchial inflammatory reaction is found in rats treated with the vehicle and corn oil. Severe septal and bronchial inflammation and the increased presence of collagen (blue) are seen in bleomycin treated rats on the corn oil diet. Inflammation and vascular thickness are less severe when the diet is flaxseed oil. Stain: Masson's Trichrome, 200X. (b) Histological sections of rat lungs of the four groups sacrificed at day 7. The VC-treated rat lungs show modest septal inflammation and a mild increase of adventitial edema and fibrosis. No damage was observed in VF treated rats. Bleomycin treated rats on the corn oil diet present diffuse inflammation and septal thickening, severe vascular thickening with markedly decreased lumen patency and bronchitis with increased presence of collagen (blue). Bleomycin treated rats on the flaxseed oil diet had less severe damage with not so prominent septal thickness, more patent arterial lumina and less fibrosis. Stain: Masson's Trichrome, 200X. (c) Histological sections of rat lungs of the four groups sacrificed at day 21. The vehicle and corn oil caused diffuse septal and bronchial inflammation with increased collagen (blue) around the bronchial pulmonary arteries, and in the septa. No similar damage was observed in VF rats. Septal and bronchial inflammation is very severe in lungs of rats receiving bleomycin and on the corn oil diet, with vasculitis and fibrosis. The peribronchial arterioles were particularly involved with almost total obstruction of their lumen. Flaxseed oil diet markedly attenuated the bleomycin-induced damage both as septal inflammation and vasculitis. While the lungs of bleomycin + corn oil-treated rats were similar at 7 and 21 days, the lungs of bleomycin + flaxseed oil-treated rats were markedly improved at 21 days versus those of rats sacrificed after 7 days. Stain: Masson's Trichrome, 200X. Abbreviations used: VC (vehicle, corn oil), VF (vehicle, flax seed oil), BC (bleomycin, corn oil), and BF (bleomycin, flax seed oil).

It is evident that bleomycin-induced damage starts already in the bronchi and septa at 48 hours and progresses throughout the experiment. At day 7, the presence of collagen and vasculitis is already severe, and the damage persists up to 21 days substantially unchanged (Figures 2, 3(a), 3(b), and 3(c)). On the other hand, a strong protective effect is exerted by the flaxseed oil diet. 

**Figure 4:**
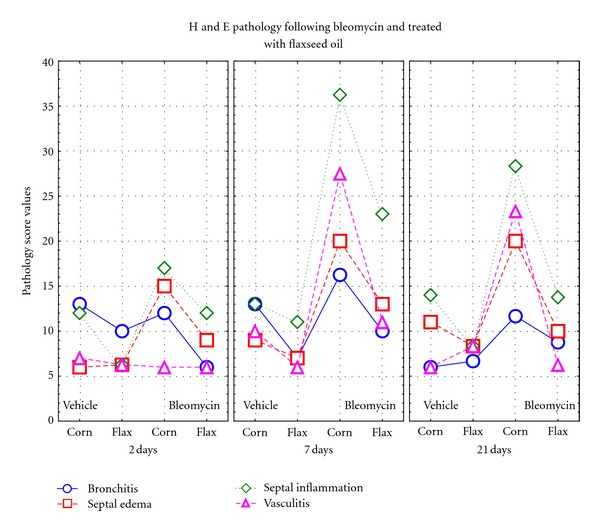
Qualitative evaluation of the pathological scores of the various treatment groups at each time interval point. (Staining: H&E). The symbols represent the mean of all the rats in each group.

**Figure 5:**
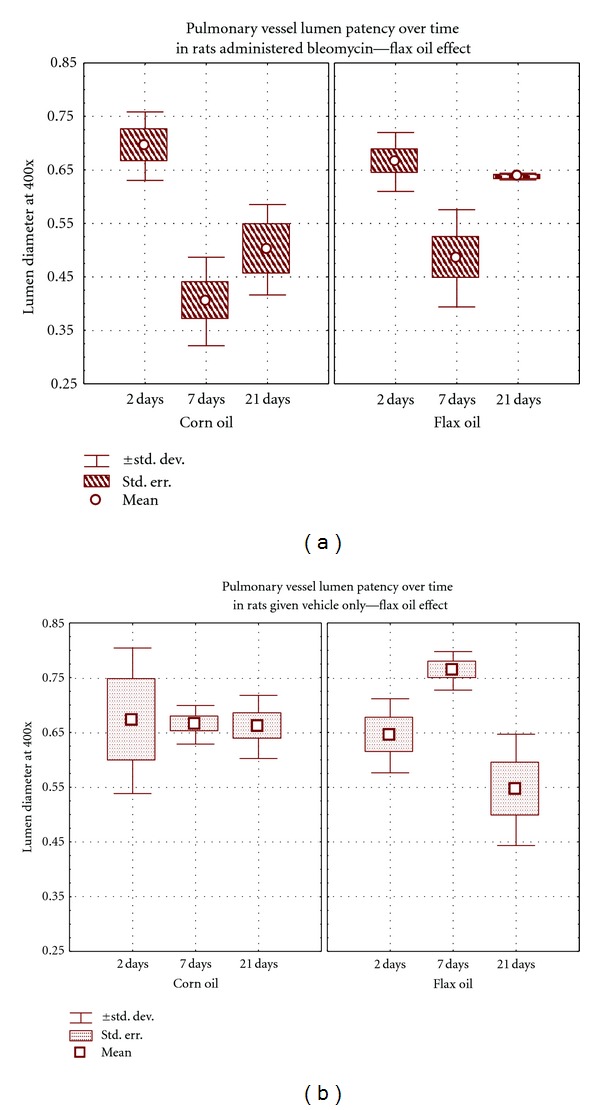
Lumen patency of small caliber pulmonary arteries and arterioles of the four groups at each time interval point. BC-treated rats show marked wall thickening and reduced lumen patency at day 7 and 21. No significant differences were observed in the lumen patency of arteries of rats instilled with the vehicle. The symbols represent means, boxes represent standard error, and the whiskers represent standard deviation values.

It has to be noted that some pulmonary damage as septal inflammation and bronchitis is also present in the rats receiving the vehicle and corn oil diet (Figure 4). The flaxseed oil diet protects the bronchi and the lungs also from the vehicle-induced inflammatory effect (Figure 5). Only bleomycin causes severe vasculitis indicating that this compound presents strong similarities with other models of damage of this organ: exposure to radiation [38] or hypoxia [39] and administration of monocrotaline [40]. 

### 3.6. Lung Histological Results

In general, dietary corn oil and flaxseed oil treatment had no significant or severe morphological damages on the lungs of the rats without the bleomycin treatment as shown by H&E staining (Figure 4). It is interesting to note that we found a small inflammatory effect in the vehicle control on day 2 immediately after saline administration. Bleomycin treatment was evaluated by the presence of septal edema and inflammation, medial and adventitial vasculitis, bronchitis, lumen patency changes, and septal thickness. The BF group showed less severe edema formation (*P* = 0.025), a decrease in septal inflammation (*P* = 0.04), and a decrease in vasculitis (*P* = 0.007) compared to the BC group (Figure 4). Differences were more significant at days 7 after bleomycin treatment than at day 2.

Furthermore, five small caliber pulmonary arteries of each rat in each group were evaluated for bleomycin-induced pulmonary vasculitis, by measuring pulmonary lumen patency and the ratio of media diameter/adventitia diameter. At days 7 and 21, bleomycin produced a significant reduction in pulmonary lumen patency (*P* = 0.01) but not of the media/adventitial diameter. However, this significant reduction of the lumen patency was not evident when the rats were on the flaxseed oil diet (Figure 5). Also, the BF group showed a significantly reduced pulmonary septal thickness at day 7 and 21 compared to the BC group (*P* = 0.013) (Figures 3(b), and 3(c)). 

The histological changes induced by bleomycin and the two diets observed with H&E staining were reinforced and further supported by the trichrome staining (Figures 3(a), 3(b), and 3(c)) of septal and bronchial inflammatory vasculitis. Increased presence of peribronchial, perivascular, and interseptal collagen was well evident in BC rat lungs, especially at days 7 and 21. The flaxseed oil diet reduced the damaging effect of bleomycin especially in rats sacrificed at 21 days. A small bronchial and septal inflammatory response was also observed in VC rat lungs, but not in VF rat lungs.  Figure 2 summarizes the histopathological changes observed with the trichrome staining of the lungs. The trichrome data supports the previously reported H&E information at the histological evaluation.

Histological data in the lungs of rats sacrificed 48 hours after bleomycin instillation already indicate that both bleomycin and the vehicle produce varied degrees of bronchial damage and septal inflammation with the corn oil diet; damage is less evident in the animals fed the flaxseed oil diet. After 7 days, the bleomycin-induced damage is very severe without signs of improvement two weeks later. At both interval times however, the flaxseed oil diet significantly mitigates the bleomycin-induced effects and also the minor damaging effect of the vehicle (Figure 2). The flaxseed oil effect is particularly evident on the hyperproduction of collagen, thus protecting the organ from one of the most severe side effects of bleomycin as an antineoplastic drug: the onset of organ fibrosis. 

## 4. Discussion

The purpose of this study was to determine whether dietary flaxseed oil prevented bleomycin-induced lung fibrosis. These results indicate that flaxseed oil was effective in protecting lung tissue from bleomycin-induced pulmonary toxicity in rats indicated by increased lumen patency and reduced pulmonary septal thickness, decreased inflammatory cell infiltrate, delayed edema formation, reduced vasculitis, and pulmonary and peribronchial fibrosis. 

To our knowledge, the present study is the first evidence that flaxseed oil prevents lung fibrosis using a bleomycin rat model. In the present study, we observed the short chain fatty acids in flaxseed oil to be equally effective in protecting against fibrosis as long chain fatty acids in past studies [17, 18]. Others have shown that dietary flaxseed decreases lung fibrosis and inflammation in the lungs of mice at four months after X-ray-radiation-therapy-(XRT-)induced pneumonopathy [41].

A potential mechanism to explain the anti-inflammatory role of flaxseed oil involves lung eicosanoid production. Long chain omega-3 fatty acid (EPA+DHA) eicosanoid derivatives are known to produce less active proinflammatory products than those of the typical omega-6 eicosanoid precursor, arachidonic acid [42], and in a previous work, we have showed that fish oil (EPA+DHA) protects against lung inflammation and pulmonary fibrosis in a monocrotaline-induced lung fibrosis model [17]. In addition, ALA derivatives have also been shown to have some anti-inflammatory action by competitively inhibiting the transformation of arachidonic acid to leukotrienes [43, 44]. Furthermore, ALA decreases production of the profibrotic PGE-2 series precursor, arachidonic acid [45]. Also, a bleomycin-induced lung fibrosis study in hamsters found that the omega-6 fatty acid *γ*-linolenic acid inhibited fibrosis, via its elongation in vivo to dihomo-*γ*-linoleic acid with little formation of arachidonic acid, suggesting a potential role for eicosanoid metabolites [20]. 

Another potential protective mechanism of flaxseed oil against bleomycin-induced pulmonary fibrosis may include specific cytokines. The presence of platelet-derived growth factor (PDGF) has been observed in the bleomycin model of pulmonary fibrosis [46]. The expression of PDGF was elevated in the early stage of disease and reached its peak at day 7 in bleomycin treated groups [46]. In addition, the gene expression of another profibrotic cytokine, transforming growth factor beta-1, has been previously found to be elevated around day 6 in the rat bleomycin model [8]. Similarly, we found pulmonary injury to be most severe at day 7, and also the difference due to flaxseed oil to be great at day 7 and even greater at day 21 (Figures 2, 3(b), and 3(c)). 

Bleomycin is not the only compound eliciting in the lungs and other organs, specifically the kidneys, inflammation, and vasculitis and eventually leading to fibrosis. Strong analogies are observed with other models of experimental lung injuries causing similar histopathological damage such as exposure to radiation [38] or hypoxia [39], administration of monocrotaline [40], or lung fat embolism consequent to injection of triolein [37]. In all of these models, the renin-angiotensin system is involved, and treatments with angiotensin converting enzyme inhibitors (Captopril) [47, 48] or angiotensin type 1 receptor blockers (Losartan) [49–53] are effective. Another potential mechanism by which flaxseed oil prevents fibrosis in the bleomycin model may be by the angiotensin pathway. In preliminary and unreported data, we recently found a decreased production of angiotensin peptides in the flaxseed oil treated rats. The role of other components of the respiratory system such as clara cells, osteopontin, angiotensin peptides, smooth muscle actin, mast cells, and different cytokines should also be evaluated in the response to the treatment of flaxseed oil, especially in lieu of the observation that one of the most efficient protectors in the above-mentioned models of damage, Captopril, is also a powerful antioxidant [54]. Defining the precise mechanism for the flaxseed oil-mediated protection against bleomycin-induced pulmonary fibrosis remains an intriguing question for future studies. 

It is important to note because the conversion of ALA to longer chain omega-3 fatty acids is typically less efficient in humans than in rats, the effects of ALA-fibrotic protection via formation of longer chain omega-3 fatty acids cannot be ruled out in the rat model [55]. There does remain considerable uncertainty as to the extent to which short chain ALA is converted into the longer chain EPA and DHA in humans. Some have reported that ALA's conversion into long chain derivatives is limited in humans [55, 56], whereas others have found ALA supplementation to increase the amount of EPA [57, 58]. Future fatty acid tissue analysis should help to define the fatty acid metabolites involved.

The pathological effects of bleomycin did not appear to be due to malnutrition prior to bleomycin treatment, because there was no difference in the average weight gain across groups due to the source of dietary fat. The significant loss of body weight in bleomycin treated groups (BC and BF) after bleomycin administration suggests that the animal is responding to the toxicity (Figure 1). When flaxseed oil was administered along with bleomycin, the average weight loss was significantly reduced and paired with a greater food intake, indicating the protective effects on health maintenance of flaxseed oil over corn oil (Table 1). 

It is important to note that in this study we adopted an oropharyngeal delivery of bleomycin that has proven to be an effective way to administer bleomycin. A previous study has shown that the oropharyngeal delivery of bleomycin creates a more representative human fibrotic lung disease than traditional intratracheal instillation [32]. There was, however, a slight inflammation in the vehicle control via the oropharyngeal delivery of saline likely due to a “water boarding” effect (Figure 3(a)). As noted in the methods, we administered on average 400 *μ*L of saline into the lungs, which could be the cause of this slight inflammation. Interestingly, the flaxseed oil appeared to protect also against this “water boarding” effect (Figures 3(a), 3(c), and 5).

Lastly, this study was able to show a protective effect of flaxseed oil against the bleomycin-induced fibrosis within only a four-week period of dietary treatment. This apparently provides sufficient time for the omega-3 fatty acids to become incorporated into the phospholipid membranes [17]. The four weeks of dietary treatment was half the length of time of dietary treatment that previously showed a protective effect of fish oil against the fibrosis [18]. This difference in feeding time may have significant implications in considering testing the protective effects of omega-3 fatty acids against fibrosis in the clinical setting.

## 5. Conclusions

In summary, our results provide first evidence that dietary flaxseed oil decreases inflammation and protects against lung fibrosis in bleomycin-treated rats. These results suggest that ingestion of dietary omega-3 fatty acids may provide an effective protection plan for the antineoplastic drug bleomycin by reducing the deleterious side effect of pulmonary fibrosis.

## Figures and Tables

**Table:** (a)

	VC	VF	BC	BF
2 days	0.6 ± 2.3^a^	0.8 ± 1.9	−17.5 ± 5.3	−20.7 ± 1.6
7 days	23.6 ± 2.2	31.0 ± 1.8	−71.7 ± 2.5	−37.6 ± 15.4*
21 days	97.3 ± 1.7	88.4 ± 11.9	4.2 ± 62.4	16.9 ± 31.0

Rat weights measured each week, number of rats: *n* = 3–5.

^a^Data are expressed as mean ± SEM. The numerical values are expressed as grams (g).

Significant differences are noted in a row for the respective measurement. The level of significance is *P* < 0.05.

*BC versus BF groups, *P* < 0.03.

**Table:** (b)

VC	VF	BC	BF
23.0	22.2	3.4	11.7^∗,a^

Food intake measured daily, number of rats: *n* = 13–18.

^a^Data are expressed as mean ± SEM. The numerical values are expressed as grams (g).

Significant differences are noted in a row for the respective measurement. The level of significance is *P* < 0.05.

*BC versus BF groups, *P* < 0.001.

**Table 2:** Relative (normalized to body weight) weights (g) of organs at 7 days after bleomycin treatment and averaged by experimental group.

7 days	VC	VF	BC	BF
Lung	4.3 ± 0.1^a^	4.0 ± 0.1	15.5 ± 0.1	10.3 ± 1.0*
Heart	4.9 ± 0.1	4.2 ± 0.3	5.5 ± 0.1	4.6 ± 0.2*
Liver	33.0 ± 0.6	32.7 ± 0.2	37.8 ± 1.9	32.3 ± 0.8*
Left kidney (LK)	4.1 ± 0.1	4.2 ± 0.1	5.3 ± 0.2	4.6 ± 0.1*
Right kidney (RK)	4.1 ± 0.1	4.2 ± 0.1	5.4 ± 0.1	4.8 ± 0.1*

Organ tissue weights measured at the time of sacrifice, number of rats: *n* = 3–5.

No significant differences were found at days 2 and 21.

^a^Data are expressed as mean ± SEM; all values to 10^−3^.

Significant differences comparing BC and BF groups are noted in a row for the respective tissue.

*BC versus BF groups, *P* < 0.05.
